# Pharmacokinetics of immediate release, extended release, and gastric retentive gabapentin formulations in healthy adults

**DOI:** 10.5414/CP203166

**Published:** 2018-04-10

**Authors:** Dennis Swearingen, Gerald M. Aronoff, Sabrina Ciric, Ritu Lal

**Affiliations:** 1 Banner Health, Phoenix, AZ,; 2 Carolina Pain Associates PA, Charlotte, NC, USA,; 3 Celerion, Montreal, QC, Canada, and; 4 Consultant for Arbor Pharma, Atlanta, GA, USA

**Keywords:** postherpetic neuralgia (PHN), gabapentin, gabapentin enacarbil, pharmacokinetics

## Abstract

Objective: Gabapentin immediate release (GBP-IR), gabapentin gastric retentive (GBP-GR), and the prodrug gabapentin enacarbil extended release formulation (GEn) have been approved for management of postherpetic neuralgia (PHN) in adults. This is the first pharmacokinetic (PK) comparison of all three formulations using FDA-recommended doses for PHN. Materials: This study compared the steady-state PK of GBP-IR 600 mg t.i.d., GBP-GR 1,800 mg q.d., and GEn 600 mg b.i.d. in healthy adults. Methods: The open-label study consisted of a 3-day lead-in of escalating doses of GBP-IR, 5 days of treatment with each formulation (GPB-IR, GPB-GR, and GEn), and a 7-day taper period on 600 mg GEn q.d.. Plasma concentrations were collected on day 5 for each formulation. PK parameters were estimated from plasma concentration data. Results: 14 healthy subjects (7 men, 7 women; mean (SD) age, 46.8 (7.60) years; mean (SD) body mass index, 26.7 (1.7) kg/m^2^) received all doses and completed the study. GBP-GR resulted in substantially (~ 4-fold) higher peak-to-trough ratio and percent fluctuation compared to GEn. GEn resulted in more sustained and less fluctuating daily exposure relative to GBP-IR, particularly at the end of 24 hours of dosing. In contrast, gabapentin fluctuation from GBP-IR consisted of 3 distinct peaks. After dose normalization, gabapentin exposure with GEn was ~ 2.2-fold and ~ 1.4-fold higher compared to GBP-GR and GBP-IR, respectively. All treatments were well tolerated. Conclusion: GEn requires less frequent dosing compared with GBP-IR and fluctuates less with sustained gabapentin exposure throughout the day. These PK differences may have clinically relevant implications.

## Introduction 

Postherpetic neuralgia (PHN) is the most common chronic complication of herpes zoster [1]. Management of PHN often requires a combination of approaches. Treatments include topical agents, opioids, tricyclic antidepressants, and antiseizure medications in the gabapentinoid category [2]. Gabapentinoids are derivatives of γ-aminobutyric acid (GABA) and stabilize abnormal electrical activity by selectively blocking voltage-dependent calcium channels on the dorsal root ganglion, thereby reducing pain [3]. 

Approved for management of PHN in adults are three gabapentinoids which differ in their dosing regimens: Neurontin^®^, Pfizer, Inc., New York, NY, USA (gabapentin immediate release (GBP-IR); maximum dose of 1,800 mg/day in 3 divided doses i.e. 600 mg 3 times daily (t.i.d.)) [4], Gralise^®^, Depomed, Inc., Newark, CA, USA (gabapentin gastric retentive (GBP-GR); 1,800 mg once daily (q.d.)) [5], and Horizant^®^, Arbor Pharmaceuticals, LLC, Atlanta, GA, USA (gabapentin enacarbil (GEn extended release); 1,200 mg/day in two divided doses i.e. 600 mg twice daily (b.i.d.)) [6]. While the first two are formulations, the third is a prodrug of gabapentin, and all three dose regimens aim to provide adequate systemic exposure of gabapentin based on their pharmacokinetic (PK) attributes. However, the differences in PK attributes may affect the required systemic exposure for treatment of diurnal pain of PHN. 

PHN tends to increase throughout the day, peaking at ~ 8:00 PM [1]. Thus, the chronobiological characteristic of PHN requires that treatment with gabapentinoids provide an adequate and sustained systemic exposure of gabapentin [1]. Therefore, the purpose of this study was to compare the systemic exposure through PK evaluation of three gabapentinoids (GBP-IR, GBP-GR, and GEn), which are commonly prescribed at the US Food and Drug Administration (FDA)-approved doses with differing regimens (600 mg t.i.d., 1,800 mg q.d., and 600 mg b.i.d., respectively) for the treatment of PHN. 

## Methods 

### Study design 

**Figure 1.**
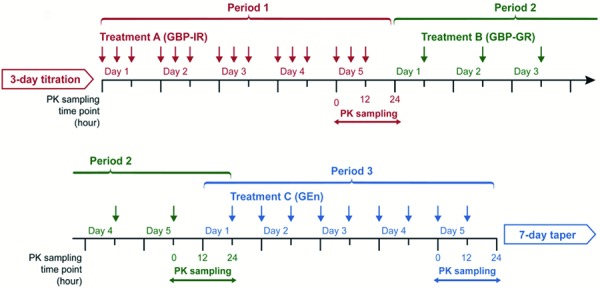
Study design. GBP-GR = gabapentin gastric retentive; GBP-IR = gabapentin immediate release; GEn = gabapentin enacarbil; PK = pharmacokinetic.

This open-label study is the first head-to-head comparison of the steady-state PK of gabapentin as GBP-IR, GBP-GR, or GEn in healthy adults. After a 3-day lead-in for dose titration of GBP-IR capsules (q.d. to t.i.d. by day 3), a 5-day treatment period for each study formulation (GBP-IR t.i.d., then GBP-GR q.d., then GEn tablets b.i.d.) was initiated, followed by a 7-day taper period during which the subjects received 600 mg GEn tablets q.d. A washout period was not required (Figure 1). Subjects were housed in the research unit from the day before titration was begun (day –4) to 24 hours after the last b.i.d. dose of GEn. Subjects returned to the research unit each morning on days 1 – 7 of the taper phase for their daily dose of GEn. On day 7 of the taper period, patient evaluation included general examination, clinical laboratory tests, a serum pregnancy test (as appropriate), and the Columbia-Suicide Severity Rating Scale (C-SSRS) questionnaire to detect emergent suicide symptoms. Safety was monitored throughout the study. 

Chesapeake Institutional Review Board in Columbia, MD, USA, approved the study, which was in compliance with the protocol, US Code of Federal Regulations, Good Clinical Practice Guidelines, and International Conference on Harmonisation tripartite guidelines. 

### Subjects 

After screening, healthy adult subjects aged 18 – 55 years, who were non-smokers with a body mass index (BMI) of 18.0 – 30.0 kg/m^2^, were enrolled. 

## Materials 

The sequential order of treatment to achieve steady state was:

- Lead-in (3 days): GBP-IR capsules (300 mg capsules, Pfizer Parke-Davis, Lot no. J83654) escalating from 1 × 300 mg to 3 × 300 mg by the third day. The reason for the fixed sequence was to allow titration (GBP-IR) and taper (GEn) to be performed with the same drug for all subjects so as to maintain consistency across subjects.
- Three treatment periods: GBP-IR tablets (600 mg tablets, Pfizer Parke-Davis, Lot no. J97053) (1,800 mg (1 × 600 mg t.i.d., approximately every 6 hours)) for first 5 days followed by GBP-GR tablets (600 mg tablets, Depomed, Inc., Lot no. MTET16681) (1,800 mg (3 × 600 mg tablets q.d. in the evening)) for the next 5 days followed by GEn tablets (600 mg tablets, XenoPort, Inc., Lot no. 3119482) (1 × 600 mg in the evening of day 1, followed by 1,200 mg (1 × 600 mg b.i.d.) from days 2 to 5) for the next 5 days.
- Taper period (7 days) with 600 mg GEn tablets q.d.

All study formulations were administered orally under fed conditions with ~ 240 mL of water and a standardized moderate-fat meal (25% – 37% calories from fat, ~ 550 kcals) as, per approved labeling, GBP-GR and GEn are required to be administered with a meal. The composition of the meal was similar during the entire study period for all formulations. 

### Sample collection 

On day 5 (steady state) of all treatment periods, blood was collected and plasma obtained for PK analysis of gabapentin using standard procedures at 0, 0.5, 1, 2, 3, 4, 5, 6, 7, 8, 9, 10, 12, 13, 14, 16, 17, 18, 19, 20, and 24 hours after the last dose of the assigned treatment. For day 1 of GBP-IR only, after completion of the titration period, blood was drawn before dosing. 

The plasma samples were analyzed for gabapentin at Celerion, Lincoln, NE, USA, using liquid chromatography/mass spectrometry. 

At the completion of b.i.d. GEn treatment, after the 24-hour PK sample collection, the C-SSRS questionnaire was repeated and urine and blood samples were collected for safety assessments. 

### Pharmacokinetic and statistical analysis 

PK parameters were calculated (Celerion, Montreal, Canada) for gabapentin in plasma, as appropriate, using non-compartmental methods in Phoenix WinNonlin^®^ (Certara USA, Inc., Princeton, NJ, USA), and are listed in Table 1. 

The actual doses used in the study were 1,800-mg equivalent for GBP-IR, 1,800 mg-equivalent for GBP-GR, and ~ 625 mg-equivalent for GEn. Therefore, the molecular weights of gabapentin enacarbil and gabapentin were taken into account for the dose normalization of PK parameters for GEn. The 1,200-mg daily dose of GEn corresponds to ~ 625 mg of gabapentin. Therefore, the PK parameters were divided by 626 mg for GEn and by 1,800 mg for GBP-IR and GBP-GR (daily doses expressed in milligrams of gabapentin). The resulting parameters were then dose normalized to a 1,000-mg dose. 

An analysis of variance (ANOVA) was performed on the natural log (ln)-transformed parameters peak-to-trough ratio, percent fluctuation ratios, area under the plasma concentration time curve (AUC_0–24_), and maximum plasma concentration (C_max_). The ANOVA model included treatment as a fixed effect and subject as a random effect. Each ANOVA included calculation of least-squares means (LSM), the difference between treatment LSM, and the standard error (SE) associated with this difference (SAS^®^ Version 9.3 MIXED procedure, Cary, NC, USA). 

Ratios of LSM were calculated using the exponentiation of the difference between treatment LSMs from the analyses on the ln-transformed parameters; 95% confidence intervals for these ratios were also calculated. 

## Results 

### Subjects 

14 subjects (7 men, 7 women) participated and completed the study per protocol (race: White, 13; Black/African American, 1; ethnicity: Hispanic/Latino, 9; Non-Hispanic/Latino, 5). The mean age (SD) was 46.8 (7.60) years (range 29 – 55), and mean BMI (SD) was 26.7 (1.7) kg/m^2^. 

**Figure 2.**
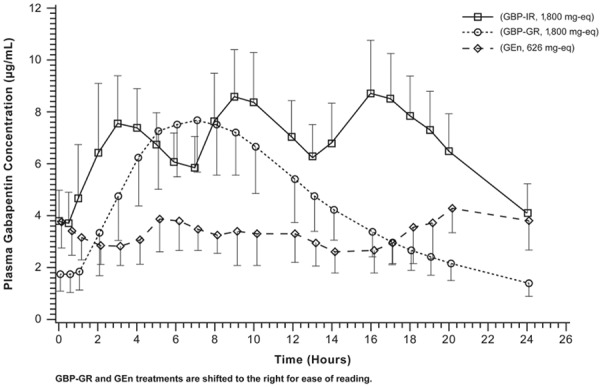
Gabapentin mean (SD) plasma concentrations versus time (linear scale) for the FDA-approved PHN doses. FDA = Food and Drug Administration; GBP-GR = gabapentin gastric retentive; GBP-IR = gabapentin immediate release; GEn = gabapentin enacarbil; PHN = postherpetic neuralgia; SD = standard deviation.

Figure 2 depicts actual plasma gabapentin concentration-time profiles over 24 hours for the three formulations. Plasma gabapentin concentration-time profiles were well characterized (Table 1). Plasma concentration-time profiles exhibited different shapes for each of the treatments. For GBP-GR q.d., a single peak was observed at 7 hours postdose, followed by a steady decline in concentration; for GBP-IR t.i.d., three distinct peaks at 3, 9, and 16 hours (the latest peak being the median time to reach maximum concentration (t_max_)) were observed; and for GEn b.i.d., sustained plasma gabapentin levels were observed throughout, and particularly at the end of the 24-hour period (Figure 2), with the median t_max_ at 18.5 hours. 

**Figure 3.**
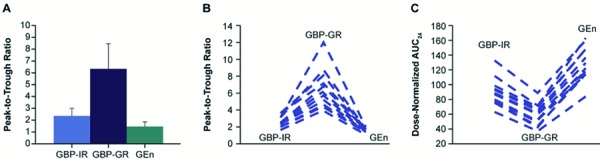
A: Plot of gabapentin dose-normalized mean (SD) peak-to-trough ratio with the three formulations (n = 14); B: Comparison of peak-to-trough ratio for individual subjects (n = 14); C: Comparison of dose-normalized AUC_0–24_ for individual subjects (n = 14). AUC_0–24_ = area under the concentration-time curve over 24 hours; GBP-GR = gabapentin gastric retentive; GBP-IR = gabapentin immediate release; GEn = gabapentin enacarbil; SD = standard deviation.

**Figure 4.**
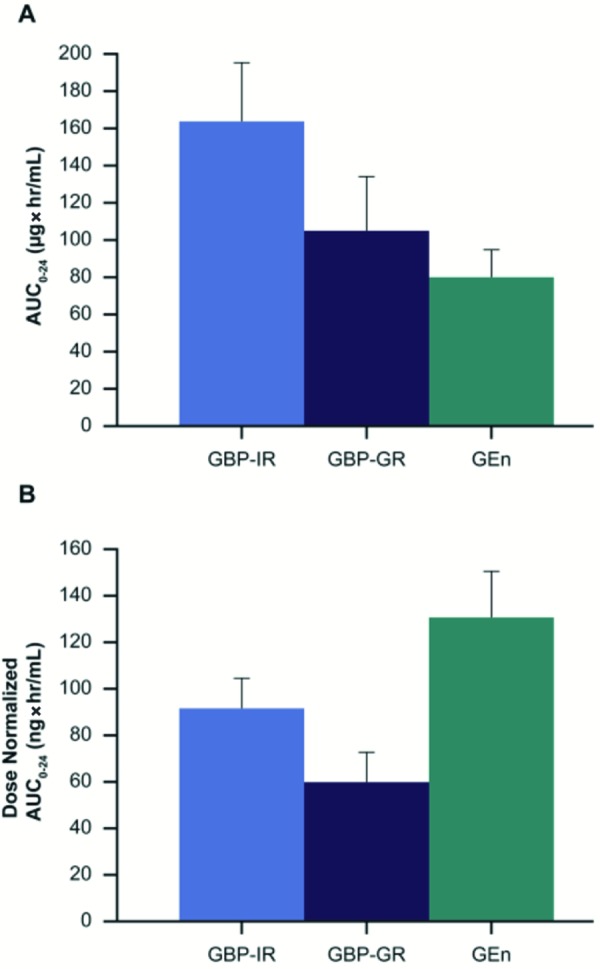
A: Plot of mean (SD) gabapentin AUC_0–24_ and B: mean (SD) dose-normalized AUC_0–24_ after dosing with the three formulations. AUC_0–24_ = area under the concentration-time curve over 24 hours; GBP-GR = gabapentin gastric retentive; GBP-IR = gabapentin immediate release; GEn = gabapentin enacarbil; SD = standard deviation.

Plasma gabapentin peak-to-trough concentration ratio and percent fluctuation were ~ 4-fold higher following GBP-GR, relative to GEn (Figure 3A, B) (Table 1). The AUC and C_max_ were 29% and 56% higher, respectively, for GBP-GR in comparison to GEn; however, following dose normalization, the AUC and C_max_ were actually 45% and 54% lower, respectively, for GBP-GR in comparison to GEn (Figure 4A, B). 

Plasma gabapentin peak-to-trough and percent fluctuation of GBP-IR were 63% and 76% higher, respectively, when compared to GEn (Figure 3A, B). The AUC and C_max_ were 2 and 1.7 times higher, respectively, for GBP-IR in comparison to GEn; however, following dose normalization, the AUC and C_max_ were actually 30% and 39% lower, respectively, for GBP-IR in comparison to GEn (Figure 4A, B). 

Plasma peak-to-trough concentration ratios were ~ 38% higher following t.i.d. dosing with GBP-IR relative to q.d. dosing with GBP-GR, with ~ 47% lower percent fluctuation (Figure 3A, B). GBP-IR resulted in a 57% higher AUC and only slightly (12%) higher C_max_ in comparison to GBP-GR for the same daily dose administered. Figure 3C provides a comparison of dose-normalized AUC for individual subjects. 

**Figure 5.**
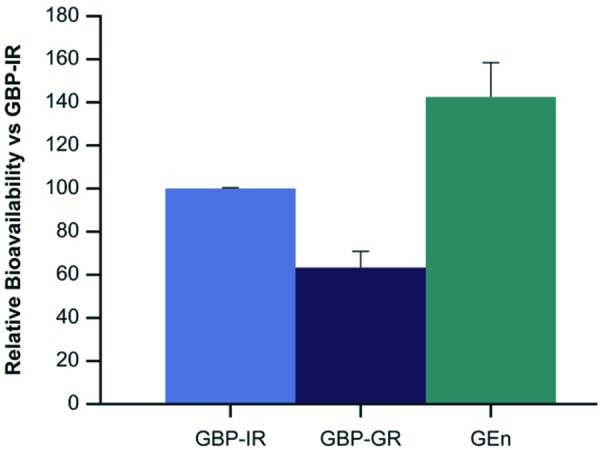
Mean (SD) relative bioavailability of GBP-GR and GEn compared to GBP-IR. GBP-GR = gabapentin gastric retentive; GBP-IR = gabapentin immediate release; GEn = gabapentin enacarbil; SD = standard deviation.

Comparisons of the peak/trough ratios between treatments and by individual subject are displayed in Figures 3A and B, respectively. Relative bioavailability of GBP-GR compared to GBP-IR was 64%. GEn had higher relative bioavailability of 143% compared to GBP-IR (Table 1) (Figure 5). 

There were no safety concerns in the study, including C-SSRS assessments. No subject experienced a serious treatment-emergent adverse event (TEAE). In total, 11 (79%) subjects reported 43 TEAEs, with > 1 TEAE reported during each stage of the study from titration through taper: 3 subjects during titration (GBP-IR capsules), 6 subjects each on GBP-IR and GBP-GR tablets, 3 subjects on GEn tablets, and 5 subjects during the taper period (GEn tablets). Forty TEAEs were mild and three were moderate in severity. Headache and somnolence were the most frequently reported TEAEs (3 subjects each, 21%). 

## Discussion 

This study examined the PK parameters of gabapentin for GBP-IR, GBP-GR, and GEn at the FDA-indicated doses, thus providing the first head-to-head controlled comparison between the three formulations. 

Of note, GBP-IR is only absorbed in the upper gastrointestinal (GI) tract and has saturable absorption. Therefore, GBP-IR has a lack of dose proportionality that decreases as the dose of gabapentin is increased. GBP-IR has a lower percentage absorbed at higher doses [7] due to saturation of transporters, and comparable gabapentin exposure between GBP-GR given q.d. or b.i.d. and GBP-IR given t.i.d. as reported by Gordi et al. [8]. 

In comparison, GBP-GR has a gastroretentive delivery formulation and is similarly only absorbed in the upper GI tract and exhibits a saturable absorption that is enhanced by high-fat foods. For example, with GBP-GR dosing, the C_max_ of gabapentin increases from 33% to 84% and AUC increases from 33% to 118% (high peak-to-trough ratio (5.2)) with higher fat content in a meal. Only with a high-fat meal does the GBP-GR formulation have highest gabapentin exposures and a delayed absorption [5, 9]. 

Both GBP-IR and GBP-GR show lack of dose proportionality due to saturable absorption. Bioavailability of gabapentin is ~ 60%, 47%, 34%, 33%, and 27% following 900, 1,200, 2,400, 3,600, and 4,800 mg/day given in 3 divided doses, respectively [4]. There are no published exposures for 900 mg GBP-IR t.i.d. dose (2,700 mg). However, from the current study, the 600-mg t.i.d. GBP-IR dose (1,800 mg) has an AUC_0–24_ of 164 µg×h/mL. So, assuming a similar bioavailability for the 1,800-mg and 2,700-mg doses (30%), the GBP-IR AUC at a 900-mg t.i.d. dose would be expected to have an AUC_0–24_ of ~ 246 µg×h/mL. However, GEn is a transported prodrug of gabapentin that seeks to overcome the PK limitations of gabapentin [10, 11]. In contrast to both GBP-IR and GBP-GR, GEn is absorbed by high-capacity nutrient transporters, such as monocarboxylate transporter type 1 and sodium-dependent multivitamin transporter, which are present throughout the intestinal tract. Therefore, the pathway for absorption of GEn is not saturated at the doses being used for treatment of PHN. Consequently, GEn achieves efficient oral absorption and conversion to gabapentin, and provides dose proportional systemic gabapentin exposure up to 6,000 mg [12] with high bioavailability [11]. 

The recommended dose of GEn is taken with food for PHN and has a low peak-to-trough fluctuation index (1.5) that indicates steady and stable exposures throughout the day [6]. 

Overall exposure (AUC) to gabapentin was slightly (29%) higher following GBP-GR compared with GEn, while maximum exposure (C_max_) was 56% higher. However, after normalizing for dose, plasma exposure to gabapentin following GBP-GR administration was ~ 50% lower in comparison to GEn. Similarly, plasma exposure following GBP-IR produced an ~ 30% lower AUC and 39% lower C_max_ compared with GEn after dose normalization. The results suggest that gabapentin exposure is more consistent and has high oral bioavailability when administered as GEn than when administered as either GBP-IR or GBP-GR. These findings are in agreement with previous observations [12, 13, 14] including Cundy et al. [14] who reported more than 2-fold higher oral bioavailability of gabapentin when dosed as GEn compared with GBP-IR. 

In this study, comparison of GEn and GBP-GR based on the ratios of geometric LSMs, plasma gabapentin peak-to-trough concentration ratios, and percent fluctuation were ~ 4-fold higher following GBP-GR relative to GEn. Exposure to gabapentin following GBP-GR had a single peak 7 hours post-dose then steadily declined throughout the rest of the 24-hour period. Comparison of the PK of GBP-IR 600 mg t.i.d. and GEn 600 mg b.i.d. showed that the peak-to-trough and percent fluctuation of GBP-IR were 63% and 76% higher, respectively, compared with those of GEn. In contrast, plasma gabapentin levels following dosing with GEn (600 mg b.i.d.) were sustained throughout the day and had lower peak-to-trough and percent fluctuation, particularly at the end of the 24-hour dosing period (Figure 3). This is in accordance with previous observations of Lal et al. [12, 13] who reported that GEn extended-release tablets provided sustained gabapentin exposure with low intersubject variability. 

Previously, Lal et al. [12, 15] also reported low intersubject variability in gabapentin exposure following dosing with GEn. Although clinicians generalize efficacy amongst these products to be similar, differences in PK attributes such as time of onset, dose proportionality, peak-to-trough ratios, and 24-hour total exposure may lead to clinically meaningful differentiation. 

The relative bioavailability of GEn was 143% compared to GBP-IR, showing that the prodrug had improved gabapentin exposure compared to GBP-IR. On the other hand, the relative bioavailability of GBP-GR was lower than that of GBP-IR (64%). Thus, a lower gabapentin equivalent dose is needed for the treatment of PHN [6], when administered as GEn (~ 625-mg equivalent), compared to when administered as GBP-IR (1,800-mg equivalent) [4] or GBP-GR (1,800-mg equivalent) [5]. 

In our study, GBP-GR was administered q.d. in the evening, with the intention of dosing that is consistent with the approved labeling recommendations. However, it is worthwhile noting that this recommended evening dose coincides with maximal pain onset, which peaks at ~ 8:00 PM and subsequently declines. Therefore, just as gabapentin exposure begins to increase with GBP-GR, pain from PHN paradoxically declines due to its well-described diurnal nature [1]. In contrast, as GEn is dosed every 12 hours and GBP-IR is dosed t.i.d., the time of maximum pain onset may be covered by GEn and GBP-IR. 

The study limitations include the fact that the study population was of healthy volunteers, which merits consideration prior to extrapolating the results to PHN patients. Furthermore, as there is no known target gabapentin level for pain relief in PHN, correlations between efficacy and gabapentin concentration should be considered with caution. The sequence of the treatments was fixed in the study, so that each subject was titrated with the same drug (GBP-IR) and tapered with the same drug (GEn). Also, given the half-life of gabapentin is 5 – 7 hours, each treatment was predicted to reach steady state by day 5, the day of PK measurements. However, the influence of sequence effect as a confounding factor cannot be ruled out for other factors such as safety or tolerability. 

## Conclusion 

The results of this study suggest that clinicians should be mindful of the relative differences in PK properties when deciding which of the three gabapentinoids to prescribe for various conditions. GEn yields higher bioavailability, and uses an ~ 3-fold lower gabapentin equivalent dose compared to GBP-IR and GBP-GR, yielding lower peak gabapentin exposure. Taken together, these PK differences should be considered when treating PHN, particularly in light of the circadian nature of this condition. Of all the various gabapentin formulations (GBP-IR, GBP-GR, and GEn), GEn provides the most sustained, lowest fluctuating gabapentin exposures throughout a 24-hour duration. 

## Funding 

This study was funded by XenoPort, Inc., Santa Clara, CA, USA (acquired by Arbor Pharmaceuticals LLC, Atlanta, GA, USA). Research funding for the design and conduct of this study, as well as the collection, management, analysis, and interpretation of the data, was sponsored by XenoPort. The authors also thank Anne S. Packer, MA (Mark Consulting Inc, Centennial, CO, USA), funded by XenoPort, and Annirudha Chillar, MD, PhD (Cactus Communications, Mumbai, India), funded by Arbor Pharmaceuticals LLC, for writing and editorial assistance during the preparation of this manuscript. 

## Conflict of interest 

Dennis Swearingen and Sabrina Ciric do not have any conflicts of interest to declare. Gerald M. Aronoff received consulting fees, travel grants, and meeting expenses from Arbor Pharmaceuticals. Ritu Lal received funding and consulting fees from Arbor Pharmaceuticals (previously XenoPort). 

**Table 1.** Gabapentin pharmacokinetic parameters.

	GBP-IR 600 mg t.i.d. (1,800 mg-eq) (N = 14)	GBP-GR 1,800 mg q.d. (1,800 mg-eq) (N = 14)	GEn 600 mg b.i.d. (~ 625 mg-eq) (N = 14)
Pharmacokinetic parameters (units), mean ± SD			
Peak-to-trough	2.36 ± 0.60	6.32 ± 2.12	1.46 ± 0.40
Peak-to-C_min_	2.79 ± 0.75	6.54 ± 2.32	2.59 ± 0.88
Percent fluctuation (%)	75.9 ± 19.1	160 ± 28.0	44.1 ± 33.5
AUC_0–24_ (µg×h/mL)	164 ± 32.0	105 ± 25.0	80.8 ± 14.5
C_max_ (µg/mL)	9.26 ± 1.87	8.43 ± 2.24	5.36 ± 1.27
t_max_ (h)	16.0 (8.00, 17.0)	7.00 (4.00, 10.1)	18.5 (5.00, 23.9)
C_min_ (µg/mL)	3.52 ± 1.20	1.39 ± 0.50	2.18 ± 0.53
C_trough_ (µg/mL)	4.10 ± 1.15	1.43 ± 0.50	3.85 ± 1.14
%Rel bioavailability vs. GBP-IR	100	64.0 ± 7.5	143 ± 15.7
Dose-normalized (DN) pharmacokinetic parameters* (units)			
AUC_0–24__DN (µg×h/mL)	91.0 ± 17.8	58.4 ± 13.9	129 ± 23.2
C_max__DN (µg/mL)	5.14 ± 1.04	4.68 ± 1.24	8.57 ± 2.03
C_min__DN (µg/mL)	1.96 ± 0.67	0.77 ± 0.28	3.48 ± 0.85
C_trough__DN (µg/mL)	2.28 ± 0.64	0.79 ± 0.28	6.15 ± 1.82

*The dose is normalized to 1,000 mg equivalent (mg-eq) of gabapentin. t_max_ is presented as median (minimum, maximum). b.i.d. = twice daily; q.d. = once daily; t.i.d. = 3 times daily; C_max_ = maximum plasma concentration over the last dosing day; t_max_ = time to reach maximum concentration over the dosing interval in which the C_max_ was observed (if the maximum value occurred more than once in that interval, t_max_ was the first time point with that value); C_min_ = minimum concentration over the last dosing day; C_trough_ = plasma trough (predose) concentration observed at the end of the last dosing interval of the last dosing day; AUC_0–24_ = area under the plasma concentration-time curve from 0 to 24 hours post-morning dose (periods 1 and 3) or 24 hours post-evening dose (period 2) (linear trapezoidal method); %Relative bioavailability compared to GBP-IR = (AUC_24__DN GBP-GR/AUC_24__DN GBP-IR) × 100 and (AUC_24__DN GEn/AUC_24__DN GBP-IR) × 100; Peak-to-trough = fluctuation between maximum and trough concentrations, calculated as C_max_/C_trough_; Percent fluctuation = percent fluctuation over 24 hours, calculated as: ([C_max_ – C_trough_] /C_avg_) × 100 where C_avg_ was defined as the average plasma concentration at steady state and was calculated as: AUC_0–24_/24.
